# An online workshop to raise awareness of pelvic floor in track and field female athletes: a quasi-experimental study

**DOI:** 10.1007/s00404-024-07790-x

**Published:** 2024-10-25

**Authors:** Elena Vico-Moreno, Juan Carlos Fernández-Domínguez, Natalia Romero-Franco, Jesús Molina-Mula, Antonio González-Trujillo, Elisa Bosch-Donate

**Affiliations:** 1 Nursing and Physiotherapy Department, University of the Balearic Islands, Road to Valldemossa, Km 7.5, 07122 Palma, Spain; 2 Health Research Institute of the Balearic Islands (IdISBa), Palma, Spain

**Keywords:** Distance education, Pelvic floor disorders, Women, Sport, Knowledge, Habits

## Abstract

**Introduction:**

Track and field is a high-impact sport that may facilitate pelvic floor dysfunction (PFD) of females. Although increasing the information may reduce deleterious habits, the traditional workshops to date did not motivate and engage the female athletes. This study aimed to evaluate the effects of an online educational workshop about pelvic floor awareness on knowledge and habits of track and field female athletes.

**Methods:**

A total of 49 track and field athletes participated in this quasi-experimental study: 38 attended an educational workshop and 11 did not. The workshop included innovative resources, such as 3D anatomic models, practical proprioceptive exercises guided by physiotherapists, and an anonymous questions and answers section. Before and 1 month later, all the athletes fulfilled an anonymous questionnaire to assess their knowledge about urinary incontinence (UI), ano-rectal incontinence (ARI), pelvic organ prolapse (POP) and female sexual dysfunction (FSD), as well as toileting and sports habits.

**Results:**

After attending the workshop, athletes obtained higher scores in knowledge about ARI (*p* = 0.019), POP (*p* < 0.001), and FSD (*p* = 0.018) compared to baseline and athletes who did not attend it. No improvements were observed in habits and knowledge about UI (*p* > 0.05). The athletes who reached 70% of correct responses about POP had greater number of healthy habits than the rest of the athletes.

**Conclusions:**

An innovative educational workshop about pelvic floor increases knowledge of track and field female athletes but is insufficient to modify their habits. Sports and health professionals should design educational strategies to manage the most unknown topics about pelvic floor care, considering that the proposed methodology and innovative resources are effective to increase knowledge.

## What does this study add to the clinical work

**Table:** 

A 90-min workshop with innovative resources improves the level of knowledge about pelvic floor in track and field female athletes. Although an only workshop is insufficient to modify toileting and sport-related habits for its care, higher knowledge female athletes had, better habits sustained.	

## Introduction

Pelvic floor dysfunctions (PFD) are medical conditions particularly related to physical efforts [1]. Despite its multifactorial origin, the prevalence is much higher in female athletes compared to sedentary or even to physically active females [2].

When exploring potential sports exercises related to PFD, those increasing forces on the pelvic floor (PF) structures are highly related [3]. For this reason, those sports characterized by increasing the intra-abdominal pressure through jumping, resistance exercises, or continuous ground impacts, are considered “high impact sports” [4].

Among these high-impact sports, all the track and field modalities like throwing, jumping, or running may increase vertical reaction forces on the PF up to 16 times the body weight [5]. Rodriguez-López et al. 2022 observed that more than the half of Spanish high-performance female athletes suffered urinary incontinence, as the most frequent PFD [6]. Therefore, new strategies should be designed for track and field female athletes to prevent and/or manage PFD.

As conservational management, increasing the athletes’ information has been proposed as an important research line [7]. However, traditional monographs involving static and theoretical information may not engage young population like female athletes. These traditional approaches may even reduce their motivation to learn about PF or to seek healthcare in case of PFD [8].

The information and communication technologies (ICT) facilitate new educational methodologies that could increase motivation and engagement for PF knowledge and lifestyle habits [9], even for athletes without PFD. Recent studies have shown that the use of 3D realistic models [10] may increase the understanding of PF anatomy and function [8, 11].

Apart from that, adding practical content to the educational strategies is essential for maintaining attention and increasing awareness of PF among young population. This type of content would be useful to learn about PF care and function.

To the best knowledge of the authors, no studies to date have explored these innovative educational strategies to improve the knowledge and habits related to PF care in track and field female athletes, as one the most affected population regarding PFD. The aim of this study was to assess the effects of an online theoretical–practical workshop, with a realistic 3D model of PF anatomy and function, on the knowledge and habits related to PFD of track and field female athletes in Spain.

## Materials and methods

### Design

A quasi-experimental study was designed. During May and June of 2023, track and field female athletes were invited to participate in a 90-min online workshop focused on raising awareness about function and care of pelvic floor. Before and 1 month later, athletes replied to an online questionnaire to score their knowledge about pelvic floor function and inform about their habits related to its care. Sociodemographic, sports-related, and symptomatology related to pelvic floor function were also collected prior to the start of the study.

### Participants

For the sample size calculation, we considered a finite population since the National Sports Council certified 29,000 track and field licenses for female athletes at least 16 years old. According to the previous studies, it was estimated that 50% of track and field female athletes have PFD [12]. With 95% of confidence level and 80% of statistical power, it was needed a minimum sample size of 47 participants to detect a mean difference of 20% in PFD knowledge.

As inclusion criteria, female athletes should train and compete for any track and field modality and be at least 16 years old. Only were excluded those female athletes who did not have the ability to adequately understand instructions in Spanish, as the national language. All female athletes with track and field license in Spain were invited to participate. The invitation was sent by e-mail throughout regional track and field federations, teams, and/or training groups, and announced in social networks (Instagram® and X®). Before being enrolled in this investigation, all athletes were informed about the study and signed the written consent. This study was approved by the Ethical Committee of the University of the Balearic Islands (ref: 124CER19) and previously registered on clinicaltrial.gov with reference number (ref: NCT05812170; date: 2023-04-19).

### Procedures

Online questionnaire: Participants were asked to complete an anonymous online questionnaire through the Jotform® platform (San Francisco, USA). Before the start of the study, usability and proper performance of the questionnaire were verified. This questionnaire was replied by athletes before and 4 weeks after the educational workshop. Athletes were asked to add a personal code to maintain their anonymity and made it possible to compare previous and posterior responses. For the previous questionnaire, the following data were collected: (1) sociodemographic (age and educational level), (2) sports data (years of experience); (3) symptomatology related to pelvic floor function according to the occurrence of urine leakages (UL), anal leakages (AL), and pain during sexual relationships (FSD, female sexual dysfunction); (4) level of knowledge related to four domains (urinary incontinence –UI-, ano-rectal incontinence – ARI-, pelvic organ prolapse –POP-, and female sexual dysfunctions –FSD-); and (5) habits related to pelvic floor care (regarding to micturition, defecation, sport training, and diet), adapted from similar previous studies [13]. Domains of UI and POP knowledge were compounded by 12 and 8 items, respectively, selected from PIKD (Prolapse and Incontinence Knowledge Questionnaire), validated in Spanish[14]. Domains of ARI and FSM knowledge were compounded by 10 ad-hoc items because of the absence of validated questionnaire regarding these domains in Spanish female athletes. Face and content validity of these ad-hoc domains were ensured by the research team in the previous studies[13]. The level of knowledge for every domain was considered adequate when 70% of correct responses were reached, like previous studies [7]. Habits sections were dichotomized to whether a female athlete performs the behavior (“yes” = sometimes, frequently or always) or did not perform it (“no” = never o rarely), since a behavior is considered habit when it is carried out with a regular tendency or practice [15] or each habit section, affirmative responses regarding unhealthy habits were summed as a score (each unhealthy habit, as that considered damaging for pelvic floor, was equivalent to 1 point, while healthy habits were not considered in the sum), like previous studies [15]. One month later, participants responded to the same questionnaire about knowledge and habits. If they had attended the workshop, they also rated the following question: “How would you rate the utility of the workshop?”, (from 1, not useful, to 10, essential).

Workshop: It was a 90-min online educational workshop about pelvic floor functions. The workshop was run up by physiotherapists specialized in pelvic floor dysfunctions, and included: an initial presentation of the team and general organization of the workshop (10 min); explanation of pelvic floor anatomy and physiology, based on visual resources such as videos and 3D models (accessed here: https://actitudproject.es/wp-content/uploads/2023/05/index.htm) (20 min); first part of practical exercises to increase the consciousness of pelvic floor anatomy, guided by a physiotherapist expert in PFD (20 min); theoretical explanation of PFD (UI, ARI, POP, and FSD) (10 min); second part of guided practical exercises to increase the consciousness of pelvic floor structures (10 min); theoretical explanation of preventive measures to care PF related to micturition, defecation, and sports training (10 min); and Questions and Answers, using the Mentimeter™ web-based application (Stockholm, Sweden) that allowed athletes to ask anonymous questions (10 min). This structure was designed based on the previous studies [9].

### Statistical analysis

Mean and standard deviations (SD) and frequencies were reported for numerical and categorical variables, respectively. Normality of data was checked with Kolmogorov–Smirnov test. Baseline characteristics of participants were compared using independent samples of Student's t tests. Since there were no between-group differences at baseline, a two-way (group × time) repeated-measures analysis of variance (ANOVA) with the Bonferroni post hoc test was used to evaluate group by time interactions and within-group and between-group effects. Confidence interval (CI) 95% were calculated for all differences and effect sizes (ES) were obtained and interpreted by using Cohen procedures, as follows: small (*d* ≤ 0.2), moderate (0.2 > *d* ≤ 0.8), or large (*d* > 0.8) [16]. The Student’s t test was used to explore differences in the number of healthy and unhealthy habits of athletes with and without adequate level of knowledge in every domain (> 70.0% of correct responses) [7]. International Business Machines (IBM) SPSS statistics, version 24.0 (Chicago, IL, 224 USA) was used, and statistical significance was set at *p* < 0.05.

## Results

**Fig. 1:**
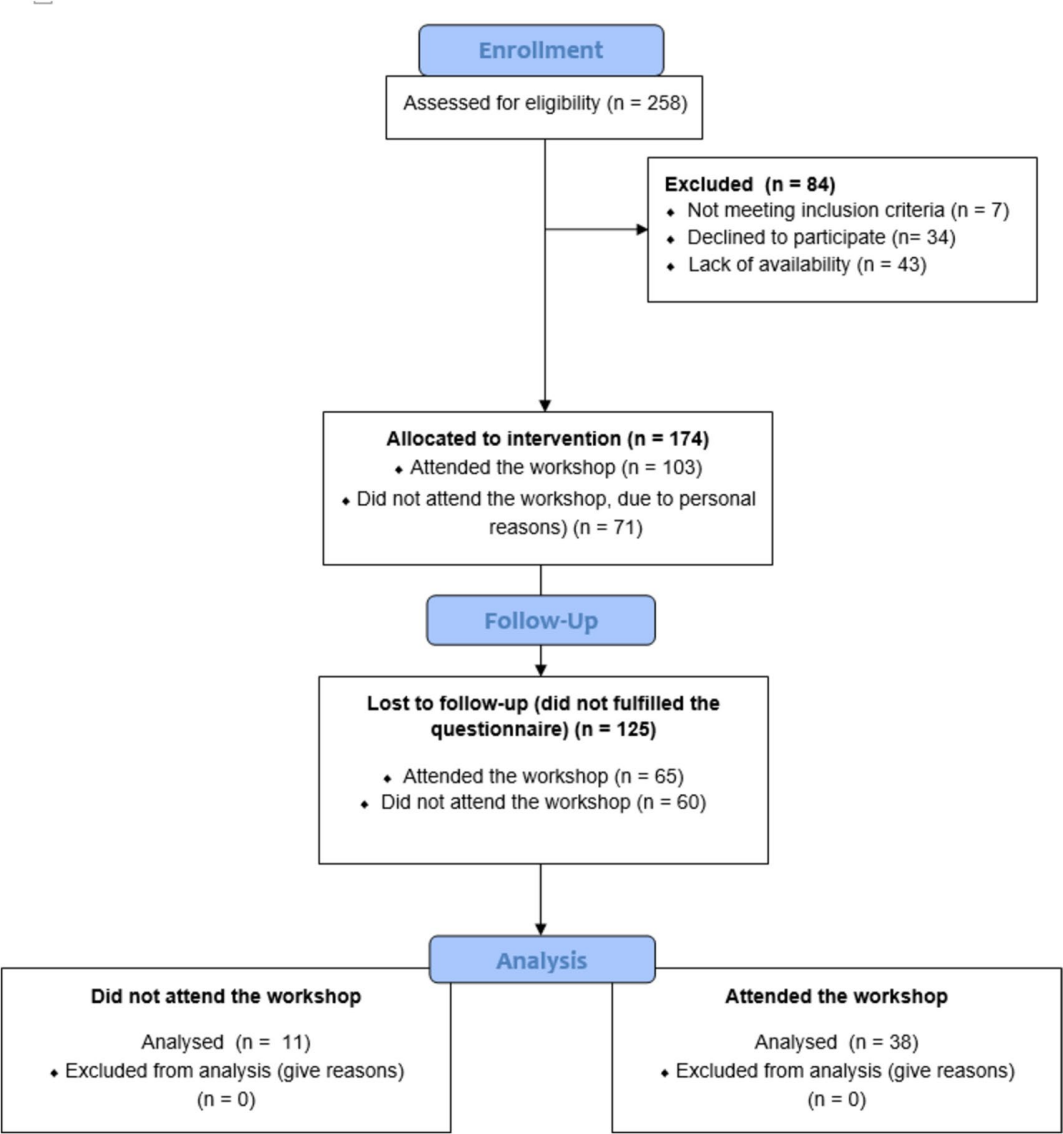
Flow diagram

A total of 174 track and field female athletes accepted to attend the workshop, but only 49 athletes completed the study (Fig. 1). The baseline characteristics of athletes who did not complete the study did not report any significant difference compared to the final participants (*p* > 0.05).

### Descriptive data

Forty-nine track and field female athletes fulfilled the questionnaire before and 1 month after the educational workshop. From them, 38 athletes attended the workshop, and 11 athletes did not. Regarding symptomatology, 25 athletes had UL (51.0%), 9 had AL (18.4%), and 33 had FSD (20.0%). The utility of the workshop was rated with 8.82 ± 1.39 points, with 27% of athletes rating with the highest punctuation (10 points). Characteristics of participants are shown in Table 1.

**Table 1:** Characteristics of the participants

	All participants (*n* = 49)
Age (years old)^¥^	35.8 (11.1)
Sports experience (years)^¥^	14.1 (9.1)
Level of academic studies (%)	
Elementary	0.0
Secondary	6.1
Medium or high grade	8.2
University degree	42.9
Master’s degree or PhD	42.9
Previous labors (%)	
Nulliparous	63.3
Vaginal delivery	32.7
Cesarean	4.1

^¥^Given as mean (standard deviation)

### Level of knowledge

Table 2 shows data regarding scores from knowledge about UI, POP, ARI, and FSD of female athletes who attended the educational workshop and did not, before and 1 month after the workshop. No significant differences were found in baseline values (*p* > 0.05). Before the workshop, 71.4% of athletes had adequate level of knowledge related to UI, 49.0% related to POP, 46.9% related to AI, and 22.4% related to FSD. These values increased up to 75.5%, 61.2%, 69.2%, and 30.6%, respectively, 1 month after the workshop. Time-by-group interactions were shown for knowledge related to POP (*p* = 0.008), ARI (*p* = 0.016), and FSD domains (*p* = 0.039). Only athletes who attended the workshop improved score compared to baseline in the three domains (*p* < 0.001, *p* < 0.001, *p* = 0.018, respectively). One month after attending the workshop, athletes also obtained higher scores compared to athletes who did not attend the workshop in knowledge related to POP (*p* < 0.001), ARI (*p* = 0.019) and FSD domains (*p* = 0.018). No differences were observed for level of knowledge of UI domain (*p* > 0.05).

**Table 2:** Knowledge about pelvic floor dysfunctions in female athletes who attended the educational workshop and do not, before and 1 month after the workshop

Knowledge domain (maximal punctuation)	Participants	Baseline		Post (one month later)		Intra-group differences (pre vs post)			Between-group differences (at post)		
		Mean	SD	Mean	SD	Mean	95% CI	ES	Mean	95% CI	ES
UI Score (12 points)	No workshop	8.64	2.38	9.09	2.02	0.45	(– 0.56 to 1.47)	NS	0.74	(0.60–2.07)	NS
	Workshop	9.40	1.58	9.83	1.89	0.43	(– 0.11 to 0.97)	NS			
POP Score (8 points)	No workshop	4.82	2.23	4.27	2.45	– 0.55	(– 1.56 to 0.47)	NS	2.13	(0.63–3.65)*	1.33
	Workshop	5.20	2.04	6.40	1.24	1.20	(0.54–1.86)*	0.71			
ARI Score (10 points)	No workshop	6.36	2.34	6.27	1.56	– 0.09	(– 1.06 to 0.88)	NS	1.24	(0.28–2.25)*	0.83
	Workshop	6.43	1.50	7.51	1.48	1.09	(0.65–1.52)*	0.72			
FSD Score (10 points)	No workshop	4.55	2.34	3.91	2.84	– 0.64	(1.60–0.33)	NS	1.86	(0.24–3.49)*	0.80
	Workshop	4.83	2.53	5.77	2.16	0.94	(0.12–1.76)*	0.64			

*ARI* ano-rectal incontinence; *CI* confidence interval; *ES* effect size; *POP* pelvic organ prolapse; *NS* non-significant; *FSD* female sexual dysfunction; *SD* standard deviation; *UI* urinary incontinence

*Significant differences *p* < 0.05

### Habits

Table 3 shows data about habits reported by athletes, before and 1 month after attending the workshop. No statistically significant within-group or between-group differences were found (*p* > 0.05).

**Table 3:** Number of habits related to pelvic floor dysfunctions in female athletes who attended the educational workshop and do not, before and 1 month after the workshop

Number of habits	Participants	Pre		Post (one month later)		Intra-group differences			Between-group differences		
						(Pre vs post)			(At post)		
		Mean	SD	Mean	SD	Mean	95% CI	ES (*d*)	Mean	95% CI	ES (*d*)
Micturition unhealthy habits (0–10)	No workshop	2.7	2.1	3.1	1.6	0.4	(0.89–1.61)	NS	1.30	(– 0.32 to 2.91)	NS
	Workshop	4.3	2.4	4.4	2.6	0.1	(– 0.53 to 0.58)	NS			
Defecation unhealthy habits (0–10)	No workshop	3.2	0.6	2.8	2	– 0.4	(– 1.50 to 0.77)	NS	0.68	(– 0.69 to 2.06)	NS
	Workshop	3.2	0.9	3.5	2	0.3	(– 0.44 to 1.07)	NS			
Sports training healthy habits (0–7)	No workshop	1.7	1.3	2.6	1.7	0.9	(– 0.27 to 2.09)	NS	0.19	(1.09–1.47)	NS
	Workshop	2.5	1.8	2.4	1.9	– 0.1	(– 0.77 to 0.57)	NS			

*CI* confidence interval; *ES* effect size; *NS* non-significant; *SD* standard deviation

### Relationships between knowledge and habits

Those athletes with adequate level of knowledge after the workshop in the POP domain also reported higher number of healthy habits related to their training routines (*p* = 0.004). No other significant relationship between level of knowledge and habits were observed (*p* > 0.05).

## Discussion

The main findings of the present study showed that a 90-min online workshop about PF with theoretical contents, 3D anatomical models, and practical exercises is useful to increase the level of knowledge related to ARI, POP, and FSD in track and field female athletes, but not for improving knowledge about UI or habits related to pelvic floor care. Female athletes who attended the workshop perceived the utility of the workshop by rating it with a very high punctuation.

Before the workshop, we observed an especially low level of knowledge in the FSD domain, but also for ARI and POP domains. Although the workshop helped to increase the level of knowledge about these domains, punctuation remained low, especially for FSD domain (57.7% as an average of correct responses after the workshop). These results were in line with the other studies, such as Neels et al. in which women were aware of the continence function of the FP (56%) and organ support (44%) but only 10% were aware of the role of the FP in sexual function and therefore did not consider their dysfunction to be a PFD [17]. The study of Bosch-Donate et al., 2024 also notes that 60% of female athletes considered normal to have occasional dyspareunia [13]. Since this finding indicates that females often normalize to have pain during sexual, educational interventions should be focused on preventing these beliefs. Interestingly, the prevalence of sexual dysfunction in females under 30 years of age was higher than in females in their 30 s and 40 s [18]. Thus, it is possible that, even though the information about FSD is available throughout Internet for all females, younger females remained being underinformed. Hence, the importance of implementing educational strategies considers the innovative ways of communication for lower ranges of age. As Pizzoferrato et al. suggested, education should take place from an early age as it has been shown to increase knowledge and to prevent future PFD [9].

Contrary to results observed in the rest of domains, the level of knowledge in the UI domain was adequate prior to the workshop, with higher than 70% of correct responses. This initial punctuation made it difficult to improve after the workshop. Like our results showed, UI is the domain most known by females, probably because it is the most frequent FP dysfunction—recall that UI affects about 56% of women according to the latest studies [19] and is therefore a priority target for treatment, both clinically and educationally. It is estimated that adequate knowledge reduces the probability of developing PFD by 57% [7]. For this reason, much effort has been made in recent years to raise awareness of UI through educational programs, as evidenced by the prolific literature on the subject [20, 21]. The lack of improvement after the workshop reflected the necessity of adding more advanced content related to this domain, apart from more difficult items in the questionnaire to explore this domain.

Regarding the adhesion of habits related to pelvic floor care, we did not observe modifications after the workshop. In this sense, it is difficult to change a routine after four weeks of an only workshop. Maybe, it would be necessary to implement educational interventions for a longer period of time or to extend the post-evaluation period. In Berujon's study, 2 sessions of 1.5 h each were given (with information on anatomy, physiology, and risk factors); after 2 months, 81% of participants reported having changed their urinary habits and 60% their bowel habits [22]. In a similar study, females attended a single PF workshop and after 2 months 80% had changed (or intended to change) their voiding habits and 71% their bowel habits [9]. In both cases, the reassessment took place 2 months after the intervention, which could be the key to habit establishment. Future studies will have to consider the need to increase this time to ensure habit adoption.

Regarding the relationship between the level of knowledge and habits related to PF, those athletes who reached adequate knowledge about POP (> 70% of correct responses) had more healthy habits related to PF care during their training routine. Since POP is a little-known PFD, it is possible that those athletes who reach a high knowledge also have an advanced general knowledge about PFD and the deleterious habits related. In line with our results, Goodridge et al. found that those females who had POP were more likely to participate in exercise programs [23]. This finding highlights the relevance of increasing the level of knowledge to promote healthy habits among females.

Although we observed high occurrence of symptomatology related to PFD among athletes, this result may be consequence of a participation bias. Those female athletes with symptoms related to PFD more likely attended to the proposed workshop and completed our study. Thus, this finding should be considered with caution. Instead, epidemiological studies with higher sample size are more appropriate to report prevalence data. Since this was not the purpose of our study, we did not include the entire diagnosis scales, but only those items related to the main PFD symptomatology. This fact also makes it possible not to extend the questionnaire more.

Our investigation had limitations. First, all participants were female athletes who trained and competed in any athletics modality. Thus, our results cannot be extrapolated to females who play other sports or sedentary females. Second, our study was compounded by two non-equivalent groups, being the control group much smaller than experimental group. Instead, studies to date that explored the effects of educational online interventions often reported results from the experimental group, without control group due the great difficulty to collect responses from athletes a second time (for the posterior questionnaire), mainly if they did not attend the workshop. This same situation eases that participants were females with PFD symptoms, being an important bias in this type of studies. In our investigation, half of participants had any type of PFD symptomatology. Finally, the absence of questionnaire specifically designed for female athletes made it necessary to use ad-hoc items that explored specific aspects of sport training. Although face and content validity of these items were ensured for our research team in a previous study, it is needed to develop validated tools for female in sport.

According to the high level of utility perceived by participants regarding to the workshop, the innovative character of the educational intervention, using 3D anatomical models to support the explanations and including exercises to improve awareness of pelvic floor structures, may be appropriate to engage and motivate female athletes.

As practical applications, health professionals should consider the design of specific educational strategies with innovative resources such as 3D models and practical contents to improve knowledge related to PFD of female athletes.

We concluded that an innovative educational workshop focused on raising awareness about pelvic floor, including 3D anatomical models to support theoretical information and exercises, is useful to increase the related knowledge of track and field female athletes, but appears to be insufficient to induce changes in habits related with its care.

## Data Availability

The data that support the findings of this study are not openly available due to reasons of sensitivity and are available from the corresponding author upon reasonable request.
